# (In)Fertility and Oxidative Stress: New Insights into Novel Redox Mechanisms Controlling Fundamental Reproductive Processes

**DOI:** 10.1155/2020/4674896

**Published:** 2020-01-21

**Authors:** E. Silva, H. Almeida, J. P. Castro

**Affiliations:** ^1^Ageing and Stress Group, Instituto de Biologia Molecular e Celular, Instituto de Investigação e Inovação em Saúde, Universidade do Porto, Portugal; ^2^Unidade de Biologia Experimental, Departamento de Biomedicina, Faculdade de Medicina da Universidade do Porto, Portugal; ^3^Brigham and Women's Hospital, Harvard Medical School, Boston, USA

Fertility is the capacity to conceive and produce offspring whereas infertility is considered the failure to do so after 12 months of regular, unprotected sexual intercourse. Currently, millions of people suffer from infertility and concerns therein increase worldwide, especially in developing countries where the majority of infertility diagnosis are established [1]. It is estimated to affect 1 in 6 couples with an almost equal contribution of male or female to the number of cases [2]. Underlying causes associate with modern lifestyle patterns or disorders that include increased maternal ageing, obesity and diabetes, anxiety, alcohol consumption, smoking, and exposure to pollutants, including those acting as endocrine disruptors.

Common to all of these conditions is the excessive production of reactive oxygen species (ROS) which may result in oxidative stress (OS) if the cellular antioxidant capacity is insufficient or ineffective to counteract ROS formation. OS is believed to contribute to infertility by interfering with fundamental processes involved in reproduction, including spermatogenesis, folliculogenesis, fertilization, implantation, and placentation [3–5]. At the subcellular level, excessive ROS production dysregulates cell signalling networks and promotes oxidation of DNA, lipids, and proteins, which can ultimately lead to cellular dysfunction by functionally altering subcellular structures such as the endoplasmatic reticulum or the mitochondria, to name a few. Thus, studies focusing on causal connections between ROS, infertility, reproductive ageing, pregnancy-related pathologies, and cellular stress are of main interest.

The most significant contributor to infertility is maternal ageing, mainly because of the age-related decay in follicle number and oocyte quality. F. Timóteo-Ferreira et al. novel findings showed that specific antioxidant supplementation in mice is able to counteract age-related oxidative stress in the ovary, fibrosis, and inflammation, thus providing an effective role in delaying ovarian ageing. The uterus is also an important contributor because it must provide an adequate microenvironment for blastocyst implantation. M. Nasiadek et al. evaluated the effect of subchronic exposure to cadmium, an environmental toxicant and endocrine disruptor present in food and smoking tobacco, on rat female reproductive system. They showed persisting cadmium-mediated plasma and uterine estradiol concentration changes, oestrous cyclicity disorders, and increased lipid peroxidation in the uterus that is able to result in ovarian and uterine dysfunction leading to infertility. In fact, the possibility that an abnormal uterine microenvironment, already present before or at the time of implantation, jeopardizes fertility and increases the risk for the development of pregnancy-related complications was reviewed by S. Mendes et al. The review addressed mechanisms by which uterine factors regulate placentation, with a special focus on ROS physiological and pathophysiological role.

The possibility of improving male fertility by alleviating OS was also addressed in this special issue. The manuscript by J. Ye et al. provided new insights into the use of metformin to improve obesity-associated male infertility. Metformin beneficial effects were mediated by a reduction of ectopic lipid accumulation in the testis, reduction of OS production, and mitigation of high-fat-diet-induced injury to the blood-testis barrier, all accompanied by male fertility improvement. An interesting article (B. H. Ali et al.) addressing water pipe smoke-induced testicular toxicity and the protective effect of nootkatone, a sesquiterpenoid isolated from plants with antioxidant and anti-inflammatory properties, is also included.

This special issue contributes with original articles that highlight and unravel mechanisms by which OS promotes infertility of both male or female origin and new antioxidant approaches that may improve fertility.
